# Complete mitochondrial genome of an olive baboon (*Papio anubis*) from Gombe National Park, Tanzania

**DOI:** 10.1080/23802359.2018.1437813

**Published:** 2018-02-09

**Authors:** Christian Roos, Idrissa S. Chuma, D. Anthony Collins, Sascha Knauf, Dietmar Zinner

**Affiliations:** a Gene Bank of Primates, German Primate Center, Leibniz Institute for Primate Research, Goettingen, Germany;; b Primate Genetics Laboratory, German Primate Center, Leibniz Institute for Primate Research, Goettingen, Germany;; c Department of Veterinary Medicine and Public Health, College of Veterinary and Biomedical Sciences, Sokoine University of Agriculture, Morogoro, Tanzania;; d Work Group Neglected Tropical Diseases, Infection Biology Unit, German Primate Center, Leibniz Institute for Primate Research, Goettingen, Germany;; e Gombe Stream Research Centre, Jane Goodall Institute, Kigoma, Tanzania;; f Cognitive Ethology Laboratory, German Primate Center, Leibniz Institute for Primate Research, Goettingen, Germany

**Keywords:** Sanger sequencing, Cercopithecidae, non-human primates

## Abstract

The olive baboon (*Papio anubis*) is the most widely distributed baboon species. We report here on the complete mitochondrial genome of an olive baboon from the south-eastern edge of the species’ range from Gombe National Park (NP), Tanzania. The genome (GenBank accession number MG787545) has a length of 16,490 bp and exhibits the typical structure of mammalian mitochondrial genomes. Phylogenetically, the olive baboon from Gombe NP is most closely related to eastern *P. anubis*, northern *P. cynocephalus* and *P. hamadryas*. The data are an important addition to further clarify the phylogeography of baboons and phylogeny of papionins in general.

Baboons, genus *Papio*, are widely distributed Old World monkeys that occur over almost all sub-Saharan Africa and parts of the Arabian Peninsula. Among the six species, the olive baboon (*Papio anubis*) has the largest range and occurs from Mali and Guinea in the West to Eritrea in the East, and South to Uganda, Democratic Republic of Congo and Tanzania (Anandam et al. 2013). Phylogenetic studies on baboons using fragments of the mitochondrial genome revealed seven major clades that display a geographic pattern, but disagree with the six species taxonomy. Using these fragments, the branching pattern among the clades remained largely unresolved, while complete mitochondrial genome data revealed a well-supported phylogeny (Zinner et al. 2013).

We report here on the sequencing of a mitochondrial genome of an olive baboon from its southernmost range from Gombe National Park (NP), Tanzania. The skin sample (individual ID 19GNM2220916; S04°40′41″, E29°37′15″) was collected for the purpose of screening for *Treponema pallidum* infection in non-human primates and not specifically for this study. Collection was in accordance with Tanzanian and German laws and guidelines. DNA was extracted with methods outlined in Knauf et al. (2016) and the complete mitochondrial genome was PCR amplified, sequenced and assembled following methods described in Zinner et al. (2013).

The newly generated mitochondrial genome exhibits and A + T content of 56.32% and contains 13 protein-coding genes, 22 transfer RNAs, two ribosomal RNAs and the control region in the order typically found in mammals (Anderson et al. 1981).

We performed phylogenetic reconstructions by adding mitochondrial genome data of other baboons representing all species and major clades (Zinner et al. 2009, 2013) and *Theropithecus gelada* as an outgroup. Sequences were aligned with Muscle 3.8.31 (Edgar 2010) in SeaView 4.5.4 (Gouy et al. 2010), and indels and poorly aligned positions were removed with Gblocks 0.91b (Castresana 2000). A maximum-likelihood tree was generated in IQ-TREE 1.5.2 (Nguyen et al. 2015) using the optimal substitution model (TrN + I+G) as selected by ModelFinder (Chernomor et al. 2016; Kalyaanamoorthy et al. 2017) and 10,000 ultrafast bootstrap replicates (Minh et al. 2013). In the obtained tree (Figure 1), the olive baboon from Gombe NP forms a strongly supported (100% bootstrap) sister lineage to a clade consisting of eastern *P. anubis*, northern *P. cynocephalus* and *P. hamadryas*, thus, further supporting the previously detected polyphyly of olive baboons (Zinner et al. 2009, 2013).

**Figure 1.**
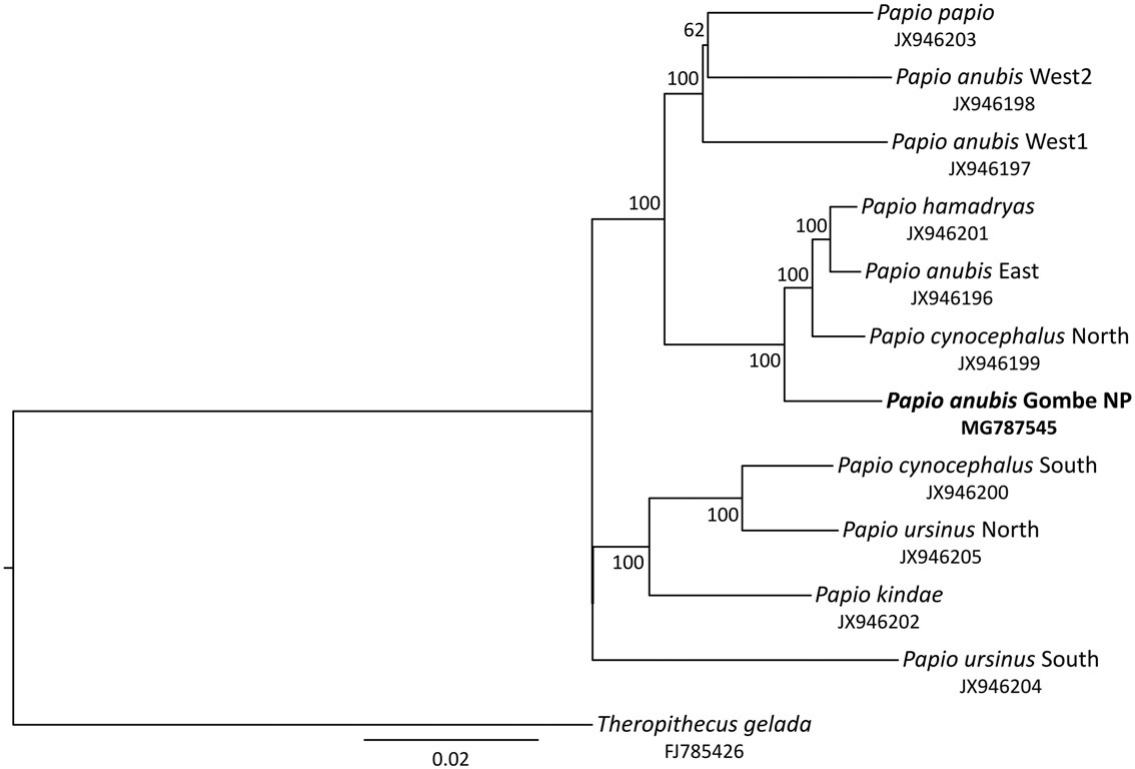
Maximum-likelihood tree showing phylogenetic relationships among baboon lineages. The olive baboon from Gombe NP (highlighted in bold) clusters with eastern *P. anubis*, northern *P. cynocephalus* and *P. hamadryas*. Numbers on nodes refer to bootstrap values and the bar indicates substitutions per site. GenBank accession numbers are listed below species.

The mitochondrial genome of an olive baboon from Gombe NP, Tanzania is an important addition to understand the phylogeography of baboons and to further investigate the phylogeny papionins in general.
